# Beetroot juice supplementation and exercise performance: is there more to the story than just nitrate?

**DOI:** 10.3389/fnut.2024.1347242

**Published:** 2024-02-20

**Authors:** William S. Zoughaib, Madison J. Fry, Ahaan Singhal, Andrew R. Coggan

**Affiliations:** ^1^Department of Kinesiology, School of Health & Human Sciences, Indiana University Indianapolis, Indianapolis, IN, United States; ^2^School of Medicine, Indiana University School of Medicine, Indianapolis, IN, United States; ^3^Indiana Center for Musculoskeletal Health, Indiana University School of Medicine, Indianapolis, IN, United States

**Keywords:** dietary nitrate, beetroot juice, nitrate salt, exercise, phytonutrients

## Abstract

This mini-review summarizes the comparative effects of different sources of dietary nitrate (NO_3_^−^), beetroot juice (BRJ) and nitrate salts (NIT), on physiological function and exercise capacity. Our objectives were to determine whether BRJ is superior to NIT in enhancing exercise-related outcomes, and to explore the potential contribution of other putatively beneficial compounds in BRJ beyond NO_3_^−^. We conducted a comparative analysis of recent studies focused on the impact of BRJ versus NIT on submaximal oxygen consumption (VO_2_), endurance performance, adaptations to training, and recovery from muscle-damaging exercise. While both NO_3_^−^ sources provide benefits, there is some evidence that BRJ may offer additional advantages, specifically in reducing VO_2_ during high-intensity exercise, magnifying performance improvements with training, and improving recovery post-exercise. These reported differences could be due to the hypothesized antioxidant and/or anti-inflammatory properties of BRJ resulting from the rich spectrum of phytonutrients it contains. However, significant limitations to published studies directly comparing BRJ and NIT make it quite challenging to draw any firm conclusions. We provide recommendations to help guide further research into the important question of whether there is more to the story of BRJ than just NO_3_^−^.

## Introduction

Initial recognition of the biological activity of dietary nitrate (NO_3_^−^) dates back to at least ancient China, where saltpeter, i.e., KNO₃, was used to treat cardiac dysfunction (1). It was not until 2007, however, that Larsen et al. (2) reported that NO_3_^−^ supplementation lowered the oxygen (O_2_) cost of submaximal exercise. Since this initial report, an extensive number of studies have examined the effects of dietary NO₃^−^ in conjunction with exercise in both healthy individuals and clinical populations, including but, not limited to, its impact on vascular function (3), muscle contractility (4), exercise economy and performance (5–7), muscle damage and pain (8), and adaptations to training (9).

Dietary NO_3_^−^ influences various physiological responses largely if not entirely by increasing nitric oxide (NO) production in the body. This occurs via an enterosalivary pathway in which NO_3_^−^ is first reduced to nitrite (NO_2_^−^) by bacteria in the oral cavity that is then further reduced to NO after absorption from the gastrointestinal tract: NO_3_^−^ → NO_2_^−^ → NO (10). NO_3_^−^ may also be reduced to NO_2_^−^ in the circulation or in the tissues themselves, via the action of, e.g., deoxyhemoglobin or xanthine oxidoreductase. Although this non-canonical pathway is normally responsible for only a small fraction of total NO synthesis (11), acute ingestion of large amounts of NO_3_^−^, i.e., 2-20x normal daily intake of ~1.5 mmol/d (12, 13), can significantly increase plasma and tissue NO_3_^−^ and NO_2_^−^ levels and hence NO production. NO is most well-known as a potent vasodilator causing blood pressure lowering effects, but in fact plays numerous other roles in physiological regulation.

NO_3_^−^ is readily available in a variety of food sources, but is mostly found in leafy green vegetables (12, 13). Beets are also high in NO_3_^−^, and in fact beetroot juice (BRJ) was first used to deliberately manipulate bodily NO_3_^−^ levels in 1984 (14). Thus, unlike the initial publication of Larsen et al. (2), who used a NO_3_^−^ salt (NIT), the vast majority of studies of the effects of dietary NO₃^−^ in the context of exercise have relied on BRJ as the source (15). This trend was magnified by the commercial production of BRJ in the form of concentrated “shots” and especially the subsequent development and validation of a NO_3_^−^-free BRJ placebo (16). Availability of this placebo greatly facilitated research in this area by permitting true double-blind experiments.

Although it is often assumed that at the same dose of NO_3_^−^ the effects of NIT and BRJ are equivalent, the results of a handful of studies tentatively suggest that BRJ might offer greater benefits during (or after) exercise than NIT (5–9). The reason for this is unclear, but it has been routinely hypothesized that other components of BRJ, e.g., polyphenols, may contribute to its effects. In other words, it is possible that the “vehicle” used to deliver NO_3_^−^ may matter. If so, such other biologically-active compounds would have to be acting in conjunction with, rather than independently from, NO_3_^−^, because NO_3_^−^-free BRJ has been found to have no effect on O_2_ uptake, muscle metabolism, or performance during exercise (17) (see Table 1).

**Table 1:** Exercise studies comparing the effects of beetroot juice (BRJ) vs. a nitrate salt.

Reference	Participants	Treatments/Treatment groups	Form of testing/Exercise	Key results	Important limitation (s)
Flueck et al. (5)	Endurance trained men (*n* = 12)	BRJ w/ NO_3_^−^ NaNO_3_ Water	Moderate and intense cycling for 5 and 8 min, respectively	6 mmol of NO_3_- from BRJ significantly reduced VO_2_ during intense exercise, but 6 mmol of NaNO_3_ did not. No changes during moderate exercise or with 3 or 12 mmol of NO_3_^−^ from either BRJ or NIT.	Inadequate blinding
Flueck et al. (6)	Upper body trained men (*n* = 14) National team paracyclists (*n* = 12)	BRJ w/ NO_3_^−^ NaNO_3_ Water	10 km handcycling time trial	No relative differences in performance with ingestion of BRJ or NaNO_3_.	Inadequate blinding
Behrens et al. (7)	Untrained men and women w/ obesity (*n* = 16)	BRJ w/ NO_3_^−^ BRJ w/o NO_3_^−^ NaNO_3_ No supplementation	Moderate and intense cycling for 3 min and to exhaustion, respectively	BRJ significantly reduced VO_2_ during moderate and increased TTE during intense exercise, but NIT did not.	Amount of NO_3_^−^ in BRJ not measured
Clifford et al. (8)	Recreationally active men (*n* = 10/group)	BRJ w/ NO_3_^−^ NaNO_3_ Isoenergetic placebo	100 drop jumps from 0.6 m	BRJ group showed improved PPT, no group differences in inflammatory markers.	Cross-sectional design
Thompson et al. (9)	Recreationally active men and women (*n* = 10/group)	BRJ w/ NO_3_^−^ KNO_3_ No supplementation	4 wk. sprint interval training	Improved with BRJ, no significant improvement with KNO_3_.	Cross-sectional design

PPT, pressure pain threshold; TTE, time to exhaustion.

Herein we review the limited number of exercise-related studies that have directly compared the effects of NIT vs. BRJ. By doing so we hope to stimulate additional research to address the intriguing, but still unanswered, question of whether BRJ has greater effects than NIT on physiological responses and/or performance during exercise.

## Studies of BRJ versus NIT with exercise

In 2016, Flueck et al. (5) were the first to report that BRJ may be superior to NIT during exercise. These authors examined the effects of acute 3, 6, or 12 mmol doses of NO_3_^−^ as BRJ or NIT on O_2_ uptake (VO_2_) during moderate and high intensity exercise. Plain water was used as a comparator. No significant differences were observed during moderate intensity exercise. During high intensity exercise, however, submaximal VO_2_ was significantly reduced at the intermediate dose when the NO_3_^−^ was provided via BRJ but not as NIT. This led the authors to conclude that BRJ may be more effective than NIT enhancing the economy of exercise, possibly by improving mitochondrial efficiency as originally proposed by Larsen et al. (18).

In contrast to the above, in a subsequent study Flueck et al. (6) found no significant effect of 6 mmol of NO_3_^−^ given acutely as either BRJ or NIT vs. plain water on VO_2_, power output, or time-to-completion of a simulated 10 km arm cycling time trial (TT) performed by paracyclists and able-bodied individuals. The ratio of power output to VO_2_ was, however, significantly higher in the able-bodied participants at several points during the TT following BRJ but not NIT, consistent with a greater improvement in cycling economy/efficiency with BRJ.

More recently, Behrens et al. (7) have also provided evidence indicating a possible difference between BRJ and NIT during exercise. These authors compared the acute effects of 6.4 mmol of NO_3_^−^ from the two sources vs. NO_3_^−^-free BRJ or nothing (as a control) in obese individuals. Although BRJ significantly reduced VO_2_ and delayed time-to-fatigue during high intensity exercise, NIT did not. Furthermore, there was a weak but significant inverse correlation between the changes in VO_2_ and changes in plasma NO_2_^−^ concentration, which was significantly higher after BRJ vs. NIT.

Based on the above results, it has been suggested that BRJ might be more effective than NIT in reducing the O_2_ cost of intense, but submaximal, exercise, thereby enhancing performance (5–7). It is unclear, however, why this might be true only at an intermediate dose of NO_3_^−^ and not at lower or higher doses (5). Furthermore, the use of plain water as a “placebo” is an obvious limitation of the studies by Flueck et al. (5, 6). Behrens et al. (7) improved on this aspect of experimental design via use of NO_3_^−^-free as well as NO_3_^−^-containing BRJ, but as pointed out by these authors it was not possible to completely blind participants to differences between BRJ and NIT.

Perhaps more importantly, although all three of these studies ostensibly provided equimolar doses of NO_3_^−^ from both BRJ and NIT, in each case plasma NO_3_^−^ (and hence NO_2_^−^) concentrations were higher following BRJ vs. NIT, sometimes by as much as 50%–100%. Behrens et al. (7) speculated that this was due to greater absorption of NO_3_^−^ of BRJ vs. NIT, due to the presence of other components in BRJ, e.g., polyphenols. However, although differences in gastric emptying of different food sources of NO_3_^−^ may contribute to a differing initial time course (19), Jonvik et al. (20) found that plasma NO_3_^−^ (and NO_2_^−^) levels were essentially identical 2–4 h after ingestion of 12.9 mmol of NO_3_^−^ provided via BRJ or NIT, i.e., over the time frame during which outcome measures such as VO_2_ are normally obtained. This is consistent with the fact that the absorption of NO_3_^−^ from either BRJ or NIT is essentially 100% (21, 22). The differences in plasma NO_3_^−^ levels reported by Flueck et al. (5, 6) and especially Behrens et al. (7) are therefore surprising and suggest the differences in VO_2_ they observed may have simply been the result of an inadvertent difference in the dose of NO_3_^−^ provided. In particular, Behrens et al. (7) did not measure the actual NO_3_^−^ concentration of the BRJ supplement provided, even though it is known to vary significantly (23). Regardless of the reason, however, interpretation of these three studies (5–7) is clouded by these differences in NO_3_^−^ bioavailability.

In a different context, Clifford et al. (8) determined the effects of dietary NO₃^−^ supplementation from BRJ or NIT on recovery from eccentric exercise, i.e., repeated drop jumps. This study was performed as a follow-up to previous investigations in which they had found BRJ to attenuate the side effects of muscle-damaging damaging exercise (24–26). Unlike in these previous studies, however, neither BRJ nor NIT mitigated the reduction in countermovement jump performance measured over 3 d following exercise induced-muscle damage. BRJ was, though, more beneficial in reducing muscle soreness than NIT or the placebo drink, both of which were matched to the BRJ for energy content via addition of maltodextrin and protein powder. This was true even though total NO_3_^−^/NO_2_^−^ concentrations did not differ between treatments. Clifford et al. (8) postulated that this may have been due to the antioxidant and anti-inflammatory properties of BRJ, even though no significant differences in various plasma markers of inflammation/muscle damage, i.e., CK, IL-6, IL-8, or TNF-α, were observed.

Finally, building on previous studies (27–29). Thompson et al. (9) have investigated whether BRJ or NIT might better modulate the physiological and performance adaptations to 4 wk. of sprint interval training (SIT) (8). Specifically, these authors hypothesized that NO_3_^−^ supplementation would help activate important signaling molecules such as PGC1α and AMPK, thus enhancing adaptations to training, but that this beneficial effect might be smaller with BRJ vs. NIT, due to the antioxidant properties of the former. Contrary to this hypothesis, SIT+BRJ actually resulted in greater increases in time-to-fatigue and VO_2peak_ than SIT+NIT or SIT alone. SIT+BRJ also reduced muscle lactate concentrations during high intensity exercise more than SIT+NIT. Finally, SIT+BRJ (and SIT alone) resulted in a greater increase in type IIa fiber percentage compared to SIT+ NIT. Thompson et al. (9) theorized that these larger improvements with SIT+BRJ may have been due to greater NO bioavailability, since plasma NO_2_^−^ declined to a greater extent during intense exercise in this trial, inferring enhanced reduction of NO_2_^−^ to NO. As hypothesized by Thompson et al. (9), this could have eased physiological strain during training, allowing the participants to train more intensely, thereby resulting in greater training-induced improvements. Submaximal VO_2_ was reduced equivalently in both SIT+BRJ and SIT+NIT groups, however, and there were no differences in muscle ATP or PCr concentrations during exercise or PCr recovery following exercise to support this hypothesis. Thus, although SIT+BRJ resulted in greater increases in exercise capacity compared to SIT+NIT or SIT alone, the mechanism responsible is unclear. An important limitations of this study is the cross-sectional nature of the design, which with only 10 participants/group means that the results could have readily been skewed by just one or two high or low “responders” to training. Furthermore, to simulate the likely practice of athletes, BRJ and NIT were administered on test days as well as during training, such that it is not possible to isolate any acute vs. chronic effects.

## Discussion

As detailed above, a handful of studies have tentatively suggested that BRJ may be more effective than NIT in enhancing various exercise-related outcomes. Assuming that such results are not simply due to differences in NO_3_^−^ dose, this implies that other compounds in BRJ must exert beneficial physiological effects. Furthermore, as indicated previously such chemicals would have to be acting in synergy with NO_3_^−^, since NO_3_^−^-free BRJ is seemingly without biological activity (17, unpublished observations). It is not entirely clear, however, what these putative component(s) of BRJ might be or precisely how they might act.

In addition to being high in NO_3_^−^, BRJ contains a variety of other nutrients, including ascorbic acid, K^+^, Mg^+^, folic acid, biotin, etc. (17, 30). Like many other plant foods, BRJ is also rich in polyphenolic compounds, including betacyanins, especially betanin (30, 31). The co-ingestion of the latter biomolecules with ascorbic acid could facilitate NO synthesis via enhanced reduction of NO_3_^−^ and/or NO_2_^−^ in the mouth or gut (32–34). However, in the studies described above differences in plasma and/or salivary NO_2_^−^ following BRJ or NIT intake have generally paralleled differences in NO_3_^−^ (5–7, 9) [Clifford et al. (8) only measured the sum of NO_3_^−^ and NO_2_^−^]. Furthermore, based on meta-analysis of the literature Siervo et al. (35, 36) have concluded that BRJ and NIT have comparable effects on blood pressure, perhaps the hallmark indicator of NO bioavailability. Differences in NO production itself from equimolar doses of NO_3_^−^ provided as BRJ or NIT therefore seem unlikely to explain the reportedly greater beneficial effects of BRJ on exercise responses.

**Figure 1:**
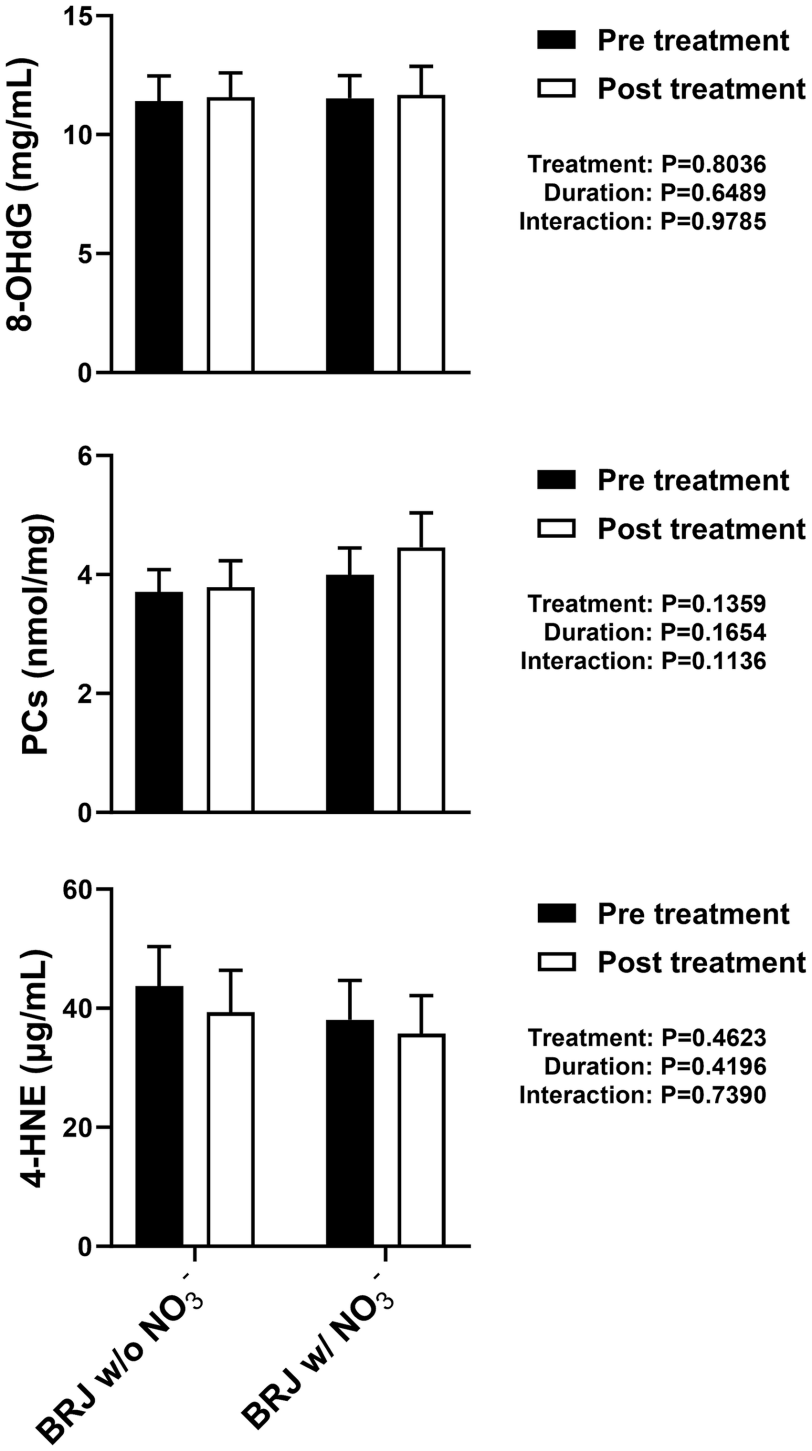
Effect of 2 wk. of daily supplementation with beetroot juice (BRJ) with or without nitrate (NO_3_^−^) on plasma markers of oxidative stress in 65–79 y old men and women (*n* = 16). 8-OHdG, 8-hydroxydeoxyguanosine. PCs, protein carbonyls. 4-HNE, 4-hydroxynonenal. No significant changes were observed. Data are redrawn from Zoughaib et al. (43).

Alternatively, rather than increasing NO production *per se* the rich concentration of polyphenols and other antioxidants in BRJ (37) could act in concert with any NO that is produced, either by prolonging NO bioavailability and/or by protecting cellular machinery from other reactive nitrogen and/or oxygen species. However, numerous studies to date have failed to reveal any influence of either acute or repeated BRJ intake on markers of oxidative stress in various populations (38–43). For example, we recently determined the effects of daily ingestion of either NO_3_^−^-containing or NO_3_^−^-free BRJ for 2 wk on plasma 8-hydroxydeoxyguanosine (8-OHdG), protein carbonyls (PCs), and 4-hydroxynonenal (4-HNE), markers of oxidative damage to DNA/RNA, proteins/amino acids, and lipids, respectively, in 65–79 y old men and women (43). No significant changes were observed (Figure 1). Although such results do not rule out a reduction in oxidative stress at the tissue level, such findings do not support the hypothesis that BRJ is more effective than NIT due to its antioxidant properties.

## Summary/conclusions/recommendations for future research

As summarized above, there are suggestions in the literature that BRJ may be superior to NIT in improving exercise-related outcomes. It is hard to make a convincing case for this hypothesis, however, due to the small number and the limitations of the studies that have been performed. More direct, head-to-head comparisons will therefore be required to definitively answer this question. To that end, we offer the following recommendations for any subsequent research in this area:

1. For any valid conclusions to be drawn, the amount of NO_3_^−^ in the BRJ and NIT supplements used must be directly measured and carefully matched. Given the wide variability in NO_3_^−^ content between different sources/lots of BRJ (23), it is not sufficient to simply rely on manufacturer’s claims [e.g., (7)].
2. Future studies should do a better job of blinding participants to the supplement being tested. For BRJ, this means comparing the effects of NO_3_^−^-containing to NO_3_^−^-free BRJ, whereas for NIT, this implies comparing, e.g., NaNO_3_ to a NaCl solution, and not to plain water [e.g., (5, 6)]. Blinding participants as to whether they are receiving BRJ or NIT is obviously more problematic, but food coloring, artificial flavoring, thickening agents, etc. could be used to help mask differences between beverages.
3. Since it is probably not possible to completely blind participants to differences between treatments, further research should initially be focused on highly reproducible physiological outcomes (e.g., VO_2_ during submaximal exercise) and not performance. If physiological responses do not differ between BRJ and NIT, there is less rationale to pursue further studies to determine possible functional differences.
4. Nonetheless, given that performance is often the key parameter of interest, researchers should consider the use of involuntary exercise, i.e., electrical stimulation protocols, as a way of circumventing possible differences in participant expectations/motivation between treatments.

Although the topic of this mini-review may seem like a trivial question, there are significant limitations to BRJ as a source of NO_3_^−^. These include issues related to cost, palatability, portability, and high levels of K^+^ and oxalate, the latter of which may preclude its use by persons with compromised renal function, e.g., the elderly, patients with heart failure. Ironically, such individuals may be the most likely to benefit from supplementation with NO_3_^−^, which can be considered a conditionally essential nutrient (44). Thus, it is important to determine whether BRJ is in fact superior to NIT for improving exercise responses. Additional studies in this area might also reveal new mechanisms or pathways by which BRJ exerts its biological effects, which could be exploited by, e.g., development of new drugs. Further research is therefore required to determine whether there is indeed more to the story of BRJ than just NO_3_^−^.

## Author contributions

WZ: Investigation, Writing – original draft, Writing – review & editing. MF: Investigation, Writing – original draft, Writing – review & editing. AS: Investigation, Writing – original draft. AC: Conceptualization, Funding acquisition, Investigation, Writing – review & editing.

## References

[1] Butler A Moffett J. Saltpetre in early and medieval Chinese medicine. Asian Med. (2009) 5:173–85. doi: 10.1163/157342109X568982

[2] Larsen FJ Weitzberg E Lundberg JO Ekblom B. Effects of dietary nitrate on oxygen cost during exercise. Acta Physiol (Oxf). (2007) 191:59–66. doi: 10.1111/j.1748-1716.2007.01713.x 17635415

[3] Craig JC Broxterman RM Smith JR Allen JD Barstow TJ. Effect of dietary nitrate supplementation on conduit artery blood flow, muscle oxygenation, and metabolic rate during handgrip exercise. J Appl Physiol. (2018) 125:254–62. doi: 10.1152/japplphysiol.00772.2017, PMID: 29722627

[4] Coggan AR Peterson LR. Dietary nitrate enhances the contractile properties of human skeletal muscle. Exerc Sport Sci Rev. (2018) 46:254–61. doi: 10.1249/JES.0000000000000167, PMID: 30001275 PMC6138552

[5] Flueck JL Bogdanova A Mettler S Perret C. Is beetroot juice more effective than sodium nitrate? The effects of equimolar nitrate dosages of nitrate-rich beetroot juice and sodium nitrate on oxygen consumption during exercise. Appl Physiol Nutr Metab. (2016) 41:421–9. doi: 10.1139/apnm-2015-0458, PMID: 26988767

[6] Flueck JL Gallo A Moelijker N Bogdanov N Bogdanova A Perret C. Influence of equimolar doses of beetroot juice and sodium nitrate on time trial performance in handcycling. Nutrients. (2019) 11:1642. doi: 10.3390/nu11071642, PMID: 31323779 PMC6683039

[7] Behrens CE Jr Ahmed K Ricart K Linder B Fernández J Bertrand B. Acute beetroot supplementation improves exercise tolerance and cycling efficiency in adults with obesity. Physiol Rep. (2020) 8:e14574. doi: 10.14814/phy2.14574 33063953 PMC7556310

[8] Clifford T Howatson G West DJ Stevenson EJ. Beetroot juice is more beneficial than sodium nitrate for attenuating muscle pain after strenuous eccentric-bias exercise. Appl Physiol Nutr Metab. (2017) 42:1185–91. doi: 10.1139/apnm-2017-0238, PMID: 28719765

[9] Thompson C Vanhatalo A Kadach S Wylie LJ Fulford J Ferguson SK. Discrete physiological effects of beetroot juice and potassium nitrate supplementation following 4-wk sprint interval training. J Appl Physiol. (2018) 124:1519–28. doi: 10.1152/japplphysiol.00047.2018, PMID: 29494294

[10] Lundberg JO Weitzberg E Gladwin MT. The nitrate-nitrite-nitric oxide pathway in physiology and therapeutics. Nat Rev Drug Discov. (2008) 7:156–67. doi: 10.1038/nrd2466 18167491

[11] Rhodes PM Leone AM Francis PL Struthers AD Moncada S. The L-arginine:nitric oxide pathway is the major source of plasma nitrite in fasted humans. Biochem Biophys Res Comm. (1995) 209:590–6. doi: 10.1006/bbrc.1995.1541, PMID: 7794389

[12] Hord NG Tang Y Bryan NS. Food sources of nitrates and nitrites: the physiologic context for potential health benefits. Am J Clin Nutr. (2009) 90:1–10. doi: 10.3945/ajcn.2008.27131, PMID: 19439460

[13] Babateen AM Fornelli G Donini LM Mathers JC Siervo M. Assessment of dietary nitrate intake in humans: a systematic review. Am J Clin Nutr. (2018) 108:878–88. doi: 10.1093/ajcn/nqy108, PMID: 30321271

[14] Ladd KF Newmark HL Archer MC. N-nitrosation of proline in smokers and nonsmokers. J Natl Cancer Inst. (1984) 73:83–7. doi: 10.1093/jnci/73.1.83 PMID: 6588238

[15] Griffiths A Alhulaefi S Hayes EJ Matu J Brandt K Watson A. Exploring the advantages and disadvantages of a whole foods approach for elevating dietary nitrate intake: have researchers concentrated too much on beetroot juice? Appl Sci. (2023) 13. doi: 10.3390/app13127319

[16] Gilchrist M Winyard PG Fulford J Anning C Shore AC Benjamin N. Dietary nitrate supplementation improves reaction time in type 2 diabetes: development and application of a novel nitrate-depleted beetroot juice placebo. Nitric Oxide. (2014) 40:67–74. doi: 10.1016/j.niox.2014.05.003, PMID: 24858657

[17] Lansley KE Winyard PG Fulford J Vanhatalo A Bailey SJ Blackwell JR. Dietary nitrate supplementation reduces the O2 cost of walking and running: a placebo-controlled study. J Appl Physiol. (2011) 110:591–600. doi: 10.1152/japplphysiol.01070.2010, PMID: 21071588

[18] Larsen FJ Schiffer TA Borniquel S Sahlin K Ekblom B Lundberg JO. Dietary inorganic nitrate improves mitochondrial efficiency in humans. Cell Metab. (2011) 13:149–59. doi: 10.1016/j.cmet.2011.01.004, PMID: 21284982

[19] James PE Willis GR Allen JD Winyard PG Jones AM. Nitrate pharmacokinetics: taking note of the difference. Nitric Oxide. (2015) 48:44–50. doi: 10.1016/j.niox.2015.04.006, PMID: 25937621

[20] Jonvik KL Nyakayiru J Pinckaers PJM Senden JMG van Loon LJC Verijk LB. Nitrate-rich vegetables increase plasma nitrate and nitrite concentrations and lower blood pressure in healthy adults. J Nutr. (2016) 146:986–93. doi: 10.3945/jn.116.229807, PMID: 27075914

[21] Wagner DA Schultz DS Deen WM Young VR Tannenbaum SR. Metabolic fate of an oral dose of ^15^N-labeled nitrate in humans: effect of diet supplementation with ascorbic acid. Cancer Res. (1983) 43:1921–5. PMID: 6831427

[22] Coggan AR Racette SB Thies D Peterson LR Stratford RE Jr. Simultaneous pharmacokinetic analysis of nitrate and its reduced metabolite, nitrite, following ingestion of inorganic nitrate in a mixed patient population. Pharm Res. (2020) 37:235. doi: 10.1007/s11095-020-02959-w, PMID: 33140122 PMC7719268

[23] Gallardo EJ Coggan AR. What is in your beet juice? Nitrate and nitrite content of beet juice products marketed to athletes. Int J Sport Nutr Exerc Metab. (2019) 29:345–9. doi: 10.1123/ijsnem.2018-0223, PMID: 30299195 PMC8512783

[24] Clifford T Bell O West DJ Howatson G Stevenson EJ. The effects of beetroot juice supplementation on indices of muscle damage following eccentric exercise. Eur J Appl Physiol. (2016) 116:353–62. doi: 10.1007/s00421-015-3290-x, PMID: 26537365

[25] Clifford T Berntzen B Davison GW West DJ Howatson G Stevenson EJ. Effects of beetroot juice on recovery of muscle function and performance between bouts of repeated sprint exercise. Nutrients. (2016) 8:506. doi: 10.3390/nu8080506, PMID: 27548212 PMC4997419

[26] Clifford T Allerton DM Brown MA Harper L Horsburgh S Keane KM. Minimal muscle damage after a marathon and no influence of beetroot juice on inflammation and recovery. Appl Physiol Nutr Metab. (2017) 42:263–70. doi: 10.1139/apnm-2016-0525, PMID: 28165768

[27] De Smet S Van Thienen R Deldicique L James R Bishop DJ Hespel P. Nitrate intake promotes shift in muscle fiber type composition during sprint interval training in hypoxia. Front Physiol. (2016) 7:233. doi: 10.3389/fphys.2016.00233 27378942 PMC4906611

[28] Muggeridge DJ Sculthorpe N James PE Easton C. The effects of dietary nitrate supplementation on the adaptations to sprint interval training in previously untrained males. J Sci Med Sport. (2017) 20:92–7. doi: 10.1016/j.jsams.2016.04.014, PMID: 27260004

[29] Thompson C Wylie LJ Fulford L Kelly J Black MI McDonagh ST. Influence of dietary nitrate supplementation on physiological and muscle metabolic adaptations to sprint interval training. J Appl Physiol. (2017) 122:642–52. doi: 10.1152/japplphysiol.00909.2016, PMID: 27909231 PMC5401949

[30] Wootton-Beard PC Moran A Ryan L. Stability of the total antioxidant capacity and total polyphenol content of 23 commercially available vegetable juices before and after in vitro digestion measured by FRAP, DPPH, ABTS and Folin–Ciocalteu methods. Food Res Int. (2011) 44:217–24. doi: 10.1016/j.foodres.2010.10.033

[31] Clifford T Constantinou CM Keane KM West DJ Howatson G Stevenson EJ. The plasma bioavailability of nitrate and betanin from *beta vulgaris* rubra in humans. Eur J Nutr. (2017) 56:1245–54. doi: 10.1007/s00394-016-1173-5, PMID: 26873098 PMC5346430

[32] Peri L Pietraforte D Scorza G Napolitano A Fogliano V Minetti M. Apples increase nitric oxide production by human saliva at the acidic pH of the stomach: a new biological function for polyphenols with a catechol group? Free Radic Biol Med. (2005) 39:668–81. doi: 10.1016/j.freeradbiomed.2005.04.021, PMID: 16085185

[33] Rocha BS Gago B Barbosa RM Laranjinha J. Dietary polyphenols generate nitric oxide from nitrite in the stomach and induce smooth muscle relaxation. Toxicology. (2009) 265:41–8. doi: 10.1016/j.tox.2009.09.008, PMID: 19778575

[34] Pereira C Ferreira NR Rocha BS Barbosa RM Laranjinha J. The redox interplay between nitrite and nitric oxide: from the gut to the brain. Redox Biol. (2013) 1:276–84. doi: 10.1016/j.redox.2013.04.004, PMID: 24024161 PMC3757698

[35] Siervo M Lara J Ogbonmwan I Mathers JC. Inorganic nitrate and beetroot juice supplementation reduces blood pressure in adults: a systematic review and meta-analysis. J Nutr. (2013) 143:818–26. doi: 10.3945/jn.112.170233, PMID: 23596162

[36] Lara J Ashor AW Oggioni C Ahluwalia A Mathers JC Siervo M. Effects of inorganic nitrate and beetroot supplementation on endothelial function: a systematic review and meta-analysis. Eur J Nutr. (2016) 55:451–9. doi: 10.1007/s00394-015-0872-7, PMID: 25764393

[37] Wootton-Beard PC Ryan L. A beetroot juice shot is a significant and convenient source of bioaccessible antioxidants. J Funct Foods. (2011) 3:329–34. doi: 10.1016/j.jff.2011.05.007

[38] Oggioni C Jakovlevic DG Klonizakis M Ashor AW Rudduck A Ranchordas M. Dietary nitrate does not modify blood pressure and cardiac output at rest and during exercise in older adults: a randomised cross-over study. Int J Food Sci Nutr. (2018) 69:74–83. doi: 10.1080/09637486.2017.1328666, PMID: 28562133 PMC5952182

[39] Carriker CR Rombach P Stevens BM Vaughan RA Gibson AL. Acute dietary nitrate supplementation does not attenuate oxidative stress or the hemodynamic response during submaximal exercise in hypobaric hypoxia. Appl Physiol Nutr Metab. (2018) 43:1268–74. doi: 10.1139/apnm-2017-0813 29775547

[40] Carriker CR Harrison CD Bockover EJ Ratcliffe BJ Crowe S Morales-Acuna F. Acute dietary nitrate does not reduce resting metabolic rate of oxidative stress marker 8-isoprostane in healthy males and females. Int J Food Sci Nutr. (2019) 70:887–93. doi: 10.1080/09637486.2019.1580683 31148492

[41] Kozlowska L Mizera O Gromadzińska Janasik B Mikołajewski K Mróz A Wąsowiscz W. Changes in oxidative stress, inflammation, and muscle damage markers following diet and beetroot juice supplementation in elite fencers. Antioxidants. (2020) 9:571. doi: 10.3390/antiox9070571, PMID: 32630279 PMC7402086

[42] Karimzadeh L Behrouz V Sohrab G Hedayati M Emami G. A randomized clinical trial of beetroot juice consumption on inflammatory markers and oxidative stress in patients with type 2 diabetes. J Food Sci. (2022) 87:5430–41. doi: 10.1111/1750-3841.16365, PMID: 36342289

[43] Zoughaib WS Hoffman RL Yates BA Moorthi RN Lim K Coggan AR. Short-term beetroot juice supplementation improves muscle contractility but does not reduce blood pressure or oxidative stress in 65-79 y old men and women. Nitric Oxide. (2023) 138-139:34–41. doi: 10.1016/j.niox.2023.05.005, PMID: 37244392 PMC10527284

[44] Pinaffi-Langley ACDC Dajani RM Prater MC Nguyen HVM Vrancken K Hays FA. Perspective: dietary nitrate from plant foods: a conditionally essential nutrient for cardiovascular health. Adv Nutr. (2023) 15:100158. doi: 10.1016/j.advnut.2023.100158, PMID: 38008359 PMC10776916

